# Supplementary material for: Associations of Birth Size with Physical and Cognitive Function in Men and Women 60 Years and Older—Systematic Review and Meta-Analysis
Source: Nutrients. 2025 Aug 8;17(16):2583. doi: 10.3390/nu17162583 (PMC12389215; doi:10.3390/nu17162583)
Supplement: Supplementary file 1 [file nutrients-17-02583-s001.zip › nutrients-3767544-supplementary.pdf]

Supplementary Materials

Supplementary Table S1. Grouping of physical outcomes

Physical function	Author	Outcomes (tests)	Exposure
Grip strength	Bleker		BW
	Kuh		BW
	Sayer	Grip strength (kg), as measured by a dynamometer.	BW
	Sayer		BW
	Ylihärsila		BW
Performance score	Bleker	SPPB[a] score	BW
	Eriksson	SFT[b] score	BW, BL, BMI
Walking	Martin	3 meter walk	BW
	Eriksson	6 minute walk	BW, BL, BMI
Chair rise	Martin	5 rises (sec)	BW
	Eriksson	Rises in 30 sec (n)	BW, BL, BMI
Balance	Martin	Losing balance (one legged) in <5 sec (yes/no)	BW
Arm curl	Eriksson	Number of times curling weight (2 kg women/3kg men) in 30 sec	BW, BL, BMI
Lower flexibility (sit and reach)	Eriksson	Distance between fingers and toes when reaching down sitting	BW, BL, BMI
Upper flexibility (back scratch)	Eriksson	Distance between middle fingers with one hand over shoulder behind back and the other one up middle of back	BW, BL, BMI
$VO2_{max}$	Salonen	UKK[c] 2 km walk test to calculate $VO2_{max}$	BW, BL, BMI
Self-reported function	von Bonsdorff	SF-36[d] Physical Functioning Scale Score	LBW
Frailty	Haapanen	Fried criteria for frailty	BW, BL, BMI

[a]Short Physical Performance Battery
[b]Senior Fitness Test
[c]Urho Kaleva Kekkonen
[d]Short Form

BW = Birth Weight, BL = Birth Length, BMI = Body Mass Index, PI = Ponderal Index, LBW = Low Birth Weight, SGA = Small for Gestational Age

Supplementary Table S2. Grouping of cognitive outcomes

Cognitive domain	Author (n)	Outcomes (tests)	Exposure
Cognitive impairment	Tuovinen (n=2)	Self reported cognitive impairment questionnaire, Dysexecutive questionnaire	BW
	Mosing (n=1)	Cognitive impairment (assessed by TELE questionnaire by phone)	BW, BL, LBW, SGA
	Skogen (n=2)	KOLT (Kendrick object learning, modified mental state exam)	BW, BL, PI
	Erickson (n=1)	Mini-mental state exam	BW
Executive function	Skogen (n= 1)	Block design test	BW, BL, PI
	Erickson (n=1)	Trail B test	BW
	Muller (n=1)	Executive function Z-score	PI
Word fluency	Krishna (n=1)	Word Fluency (measured by Animal naming test, words in 1 minute)	BW, BL, PI
	Erickson (n=1)	Word Fluency (measured by Animal naming test, words in 1 minute)	BW
	Shenkin (n=1)	Word Fluency (measured by Controlled Word Association, words in 1 minute)	BW, BL
	Skogen (n=1)	COWAT - Word Fluency (measured by Controlled Word Association, words in 1 mintue)	BW, BL, PI
Cognition	Krishna (n=2)	Global Cognition*, Composite Cognitive Score (only fully adjusted for BW)*	BW, BL, PI
	Shenkin (n=1)	General cognitive factor (g)*	BW, BL
	Skogen (n=1)	Composite score*	BW, BL, PI
	Zhang (n=1)	Risk of low cognition*	PI
Verbal memory	Krishna (n=2)	Immediate Recall, Delayed Recall (Word list memory and recall)	BW, BL, PI
	Erickson (n=1)	Immediate/delayed recall – three outcomes reported together in table 4 (the Buschke-Fuld selective reminding test)	BW
	Shenkin (n=1)	Logical memory (Logical memory subtest of Wechler memory scale)	BW, BL
	Muller (n=1)	Memory Z-score	PI
Brain volume	Muller (n=6),	Volume (MRI[a]): Intracranial, Total brain, White matter, Gray matter, Cerebral spinal fluid, White matter lesion volume<br>Memory, processing speed, executive function (6 different cognitive tests)	BW, BL, PI
	de Rooij (n=2)	Volume (MRI[a]): Intracranial, Total Brain	BW
	Franke (n=1)	BrainAGE score (MRI[a] one single value for the whole brain aging pattern)	BW, BL, PI
	Walhovd (n=3)	Cortical area, cortical thickness, cortical volume (MRI)	BW
Processing speed	Muller (n=1)	Processing speed Z-score	PI
	Skogen (n=1)	Digit symbol (modified digit symbol test)	BW, BL, PI
	Paile-Hyvärinen (n=2)	Reaction time in divided attention task, Hit rate in associate learning task	BW
Cognitive decline	Raikkonen (n=1)	Change in FDFBIA test[b] over 50 years	BW, BL, PI
	Martyn (n=1)	Difference between AH4[c] and Hill Mill test scores	BW, BL, PI
Simple attention	Skogen (n=1)	TMA (Trail A test)	BW, BL, PI
	Erickson (n=3)	Total Blessed (two items from Blessed test: World backwards and months backward), World backwards, Serial's 7 (From MMSE)	BW
Intelligence	Martyn (n=1)	AH4[c] test score	BW, BL, PI

BW = Birth Weight, BL = Birth Length, BMI = Body Mass Index, PI = Ponderal Index, LBW = Low Birth Weight, SGA = Small for Gestational Age

	Raikkonen (n=1)	FDBIA test[b] results	BW, BL, PI
	Shenkin (n=2)	RSPM (Raven's Standard Progressive Matrices), MHT (Moray House Test no. 12)	BW, BL
Premorbid ability	Shenkin (n=1)	National Adult Reading Test	BW, BL
Visual memory	Erickson (n=1)	The Heaton Visual Reproduction Test – three outcomes reported together in Table 4	BW

*As assessed by multiple tests [a]Magnetic resonance images [b]The Finnish Defence Forces Basic Intellectual Ability Test [c]Alice Heim intelligence test

BW = Birth Weight, BL = Birth Length, BMI = Body Mass Index, PI = Ponderal Index, LBW = Low Birth Weight, SGA = Small for Gestational Age

Supplementary Table 3. Excluded articles after full-text screening, along with reasons.

**Article**	**Reason**
Araújo et al. **Maternal education, anthropometric markers of malnutrition and cognitive function (ELSA-Brasil).** BMC Public Health 2014 Vol. 14 Pages 673.	Wrong population (younger than 60y/preterm)
Boots et al. **Brain activity during Stroop task performance at age 74 after exposure to the Dutch famine during eraly gestation.** Brain Cogn 2024 Vol. 177 Pages 106-162.	Wrong exposure (not birth size)
Boots et al. **Sex-specific effects of prenatal undernutrition on resting-state functional connectivity in the human brain at age 68.** Neurobiol Aging 2022 Vol. 112 Pages 129-138.	Wrong exposure (not birth size)
Bosma et al. **To what extent does IQ 'explain' socio-economic variations in function?** BMC Public Health 2007 Vol. 7 Pages 179.	Wrong population (younger than 60y/preterm)
Costa et al. **Association between birthweight and cognitive function in middle age: the atherosclerosis risk in communities study.** Ann Epidemiol 2011 Vol. 21 Issue 11 Pages 851-6.	Wrong population (younger than 60y/preterm)
de Rooij et al. **Late-life brain perfusion after prenatal famine exposure.** Neurobiol Aging 2019 Vol. 82 Pages 1-9.	Wrong exposure (not birth size)
Elwood et al. **Long term effect of breast feeding: cognitive function in the Caerphilly cohort.** J Epidemiol Community Health 2005 Vol. 59 Issue 2 Pages 130-3.	Wrong exposure (not birth size)
Gale et al. **Factors associated with symptoms of anxiety and depression in five cohorts of community-based older people: the HALCyon (Healthy Ageing across the Life Course) Programme.** Psychol Med 2011 Vol. 41 Issue 10 Pages 2057-73	Wrong outcome (not physical or cognitive function)
Haapanen et al. **Infant and childhood growth and frailty in old age: the Helsinki Birth Cohort Study.** Aging Clin Exp Res 2019 Vol. 31 Issue 5 Pages 717-721.	Wrong exposure (not birth size)
Huang et al. **Association of birthweight and risk of incident dementia: a prospective cohort study.** GeroScience 2024 Vol 46 Issue 4 Pages 3845-3859.	Wrong population (younger than 60y/preterm)
Jantunen et al. **Relationship between physical activity and physical performance in later life in different birth weight groups.** J Dev Orig Health Dis 2018 Vol. 9 Issue 1 Pages 95-101.	Wrong exposure (not birth size)
Lithell et al. **Epidemiological and clinical studies on insulin resistance and diabetes.** Ups J Med Sci 2000 Vol. 105 Issue 2 Pages 135-50.	Wrong study design/publication type (not cohort/original research)
Mikkola et al. **Healthy ageing from birth to age 84 years in the Helsinki Birth Cohort Study, Finland: a longitudinal study.** The Lancet Healthy Longevity 2023 Vol. 4 Issue 9 Pages e499-507.	Wrong outcome (not physical or cognitive function)
Nadig et al. **Morphological integration of the human brain across adolescence and adulthood.** Proc Natl Acad Sci U S A 2021 Vol. 118 Issue 14.	Wrong population (younger than 60y/preterm)
Strathearn. **Long-term cognitive function in very low-birth-weight infants.** Jama 2003 Vol. 289 Issue 17 Pages 2209.	Wrong study design/publication type (not cohort/original research)
Walhovd et al. **Neurodevelopmental origins of lifespan changes in brain and cognition.** Proc Natl Acad Sci U S A 2016 Vol. 113 Issue 33 Pages 9357-62.	Wrong population (younger than 60y/preterm)
Wiegersma et al. **Exposure to the Dutch Famine in Early Gestation and Cognitive Function and Decline in Older Age**. Nutrients 2023 Vol. 15 Issue 2.	Wrong exposure (not birth size)
Wiegersma et al. **Prenatal exposure to the Dutch famine is associated with more self-perceived cognitive problems at 72 years of age.** BMC Geriatr 2022 Vol. 22 Issue 1 Pages 176.	Wrong exposure (not birth size)

BW = Birth Weight, BL = Birth Length, BMI = Body Mass Index, PI = Ponderal Index, LBW = Low Birth Weight, SGA = Small for Gestational Age

Search Strategy

Search string for PubMed
((("Physical function") OR (("Physical Fitness"[Mesh]) OR ("Physical Fitness"))) OR (("Cognitive function") OR (("Cognition"[Mesh]) OR ("Cognition")))) AND (((((("Aged"[Mesh]) OR (elderly)) OR (aging)) OR (aged)) OR (old)) OR (geriatric)) AND ((((("Birth Weight"[Mesh]) OR ("low Birth Weight")) OR ("lbw")) OR ("prenatal undernutrition")) OR ("prenatal malnutrition")))

Search string for Scopus
("Physical function" OR "Physical Fitness") OR ("Cognitive function" OR "Cognition") AND ("Aged" OR "elderly" OR "aging" OR "old" OR "geriatric") AND ("Birth Weight" OR "low Birth Weight" OR "lbw" OR "prenatal undernutrition" OR "prenatal malnutrition")

BW = Birth Weight, BL = Birth Length, BMI = Body Mass Index, PI = Ponderal Index, LBW = Low Birth Weight, SGA = Small for Gestational Age
